# Assessing the Reliability of Automatic Milking Systems Data to Support Genetic Improvement in Dairy Cattle

**DOI:** 10.3390/ani16010001

**Published:** 2025-12-19

**Authors:** Enrico Ponzo, Riccardo Moretti, Fernando Masia, Elisa Vrieze, Paola Sacchi, Stefania Chessa

**Affiliations:** 1 Department of Veterinary Sciences, University of Turin, 10095 Grugliasco, Italy; enrico.ponzo@unito.it (E.P.); paola.sacchi@unito.it (P.S.); stefania.chessa@unito.it (S.C.); 2 Farm Management Support, Lely International N.V., 3147 PB Rotterdam, The Netherlands; fmasia@lely.com (F.M.); evrieze@lely.com (E.V.)

**Keywords:** automatic milking system, milk production traits, health-related status traits, heritability

## Abstract

This study investigates the reliability and potential genetic utility of data recorded by automatic milking systems by comparing them with official milk recording data. Analyses focused on phenotypic distributions, correlations, systematic differences, and heritability estimates for milk production and quality traits including milk yield, fat and protein percentage, somatic cell count, and electrical conductivity. Automatic milking system data and official milk recording data shared similar distributions. Correlations between the two systems were high for milk yield (r = 0.93), but moderate for fat (r = 0.52) and protein percentage (r = 0.48), and somatic cell count (r = 0.62), suggesting that while the former provides consistent data for quantity traits, quality-related ones may be less reliable. Systematic deviations between automatic milking systems and official milk recordings emerged across different lactation stages. Heritability estimates based on automatic milking system data were generally higher than the official control for production traits, supporting their use in genetic evaluations. Electrical conductivity displayed a similar heritability to somatic cell count, but its measure is insufficiently detailed and its use as an indirect indicator of udder health is not recommended. Automatic milking system data demonstrates potential for integration into genetic selection programs, although further refinement of sensor accuracy is recommended.

## Simple Summary

Automatic milking systems are rapidly spreading among dairy farms and represent a valuable source of phenotypes thanks to the continuous monitoring of numerous traits. This real-time monitoring could enable a more accurate characterization of phenotypes and, consequently, more precise breeding. In this study, we evaluate the potential of using these data for selection by estimating genetic parameters, such as heritability, both for traits related to production and resistance to mastitis. Findings suggest that milk yield and somatic cell traits measured by automatic milking systems can be reliable and other traits could gain the advantage of appropriate calibration using external data.

## 1. Introduction

Automatic milking systems (AMSs) are among the most popular automatic tools developed for dairy farms, reducing milking time and human labor and increasing management efficiency [1]. Furthermore, as AMSs are equipped with sensors that measure key milk traits (e.g., milk yield, protein and fat percentage, and somatic cell count), they enable continuous monitoring of animal health and welfare [2,3]. Repeated measurements for each cow are recorded and stored, creating a valuable database for genetic selection, since the large amount of data can improve the accuracy of genetic parameter estimation [4]. Thus, these daily records allow a shift from traditional models to daily milk yield records, which have greater precision [5]. Data collected by AMSs can be divided into two main groups, namely production- and health-related status traits. The most frequently recorded production traits are milk yield (MY), milk flow (MF), and fat and protein percentages. Numerous studies have focused on MF, estimating heritability and calculating the accuracy of the AMS-registered data compared with the data coming from the milking parlor [6,7,8]. The MF is measured by accurate sensors [6] and is related to milk quality and the health-related status of the cow [9,10]. As for the health-related status traits, AMSs are usually equipped with sensors to measure milk electrical conductivity (EC) in each quarter and could also be equipped with sensors performing somatic cell count (SCC). Both EC and SCC showed a high genetic correlation with clinical mastitis (0.65–0.80 for the former and 0.92–0.99 for the latter). Thus, both these variables were proposed as indirect indicators for the selection of animals that are more resistant to mastitis. Nevertheless, nowadays only SCC is widely used [8,11,12]. Thus, cows with continually low values of SCC are usually considered more resistant to mastitis [13,14]. Before the implementation of AMSs, estimates were obtained from periodically recorded phenotypic data, with the recording interval determined as a trade-off between accuracy and cost (e.g., monthly measurements obtained through official milk recording or quality assurance protocols). Theoretically, AMS-based estimations are expected to provide higher accuracy and resolution. Calibration issues, previously reported in scientific literature [15], remain an important issue to be addressed. Thus, to be useful for selection purposes, AMS data accuracy must be tested and their correlation with data obtained through official milk recording must be high. In addition, the heritability of AMS-registered traits should be high enough to give reliable results. The majority of the studies about heritability of production traits estimated with AMS data did not consider fat and protein percentage or content [1,6,8,15,16] except for a single study by Nixon et al. (2009), who included fat and protein yield in the data analysis [17]. Additionally, Carlström et al. (2014) estimated the correlation between milkability traits recorded using AMSs and those obtained from conventional milking parlors [6]. However, neither fat and protein content nor percentage data were included in these studies. The aim of this study was to estimate the heritability of different productive- (namely, fat percentage, protein percentage, and milk yield) and health-related (namely, SCC and EC) traits measured with AMSs in different farms in Northern Italy. Secondly, a further aim of this study was to calculate the correlation of AMS data with official milk recording data to verify if the former could be integrated with the latter to improve selection results.

## 2. Materials and Methods

### 2.1. Farms and Animals

Five commercial Holstein Friesian dairy farms that were located in northern Italy, characterized by similar environments and management techniques, and equipped with Astronaut A5s (Lely Industries N.V., Maassluis, The Netherlands) were involved in the present study. In these farms, animals were reared indoors in freestall housing systems, all equipped with ventilation systems to control temperature and humidity. The cows were fed a total mixed ration based on grass and corn silage. All farms were equipped with two AMSs. The average parity per cow was 2.2, with a maximum of 7.

Data analyzed in this study was recorded by AMSs in a time range spanning from March 2017 to July 2023. The obtained AMS dataset was composed of 1,035,311 daily records collected from 2336 cows, as detailed below. Data cleaning was performed, removing empty records and recording errors (records completely out of scale resulting from export-induced errors, like the loss of the decimal separator). We considered only records with days in milk (DIM) ranging from 5 to 305 days and daily milking events ranging from 1 to 5. Additionally, only cows with at least 10 observations each were kept in the dataset. The final pruned dataset consisted of 885,969 records from 2181 cows.

Pedigree data were also included, where available, considering three generations of animals (i.e., up to the great-grandparents of each cow). The final pedigree consisted of 4763 total animals with 874 sires. Specifically, 764 sires had daughter records (average number of daughters per sire was 3.60 ± 4.56, ranging from 1 to 63).

### 2.2. Recorded Traits

Five main milk-related traits were included in the present study: daily MY, fat percentage, protein percentage, EC, and SCC. Each trait was measured at every milking event except for the SCC, which was measured once a day when the amount was lower than 200,000 and at each milking as soon as it passed this threshold. Nevertheless, since the other variables were recorded daily, the SCCs measured at each milking were then averaged on the same frequency. Although daily averaging does not completely remove the heterogeneity due to uneven frequency of SCC measurements by AMSs, its impact was limited because threshold-exceeding days were limited due to the low incidence of severe mastitis, the rapid SCC decline in many cows, and the relatively stable values in cows showing subclinical mastitis in the selected farms. Daily MY was volumetrically measured in kilograms and was obtained as the sum of the recorded production from all the milking events in a day. Fat and protein were expressed as percentages, measured for each quarter separately and then combined into a single final value for each trait. The fat and protein sensor equipped in the AMSs mainly employs measurements of the milk’s infrared spectrum to estimate the values.

Regarding EC, two different values were considered in this study, namely the average EC from all of the milking events in a day (aEC) and the maximum EC value recorded in a single day (mEC). All sensors installed in this type of AMS are typically calibrated three times per year by specialized technicians. Lastly, to normalize the SCC data, somatic cell score (SCS) was calculated as follows:

$$SCS={log}_{2}(\frac{SCC}{100,000})+3$$

The cows’ individual data that were recorded and analyzed in this study were farm, sire and dam ID, date of birth (DOB), DIM, and parity. The age of each cow at data recording was obtained by subtracting DOB from the test day recorded by the AMS.

### 2.3. Official Milk Recording Data

The same five main milk-related variables were also obtained from the monthly milk composition analyses performed by specialized technicians from the Piedmont Regional Breeder Association of the five farms enrolled in the study. These official milk recordings (OMRs), performed by accredited laboratories, are considered gold standard measurements and analyses. A total of 33,231 observations from 2387 cows were available from the five farms, independently of how many cows were milked by the AMSs. The number of cows was higher than the one recorded by the AMSs since some farms were transitioning from milking parlor to AMSs and had cows that could not get used to robots or were still traditionally milked until culling.

### 2.4. Datasets

Three different datasets were analyzed in this study: dataset 1 consisted of all the valid or edited data recorded by the AMSs (885,969 records from 2181 cows); dataset 2 included all the OMR data (33,231 observations from 2387 cows milked by both AMS and milk parlor); dataset 3 merged data from AMSs and OMR data (19,488 observations from 1982 cows). In particular, we retained only the data from the same day as the OMR for cows that had both AMS and OMR records.

### 2.5. Statistical Analyses and Models

All the statistical analyses were performed in an R software environment v4.2.3 [18]. Regarding dataset 1, the correlation between different traits measured by AMS was calculated.

To check for measurement differences due to the different techniques employed (i.e., AMS sensors versus laboratory analyses), the correlation between the same trait measured by AMS and OMR was calculated for dataset 3. Furthermore, the heritability values of each milk-related variable were estimated separately for primiparous and multiparous cows using restricted maximum likelihood (REML) through the remlf90 function of the R package breedR v0.12-5 [19]. The function remlf90 allows for fixed effects, random effects (including additive genetic, spatial, and generic covariance structures), and optional model components. The models used included both an additive genetic effect and a permanent environmental effect as random effects and DIM, number of milkings/day, age at last calving, and parity (only in the multiparous subset) as fixed effects. Milk yield, fat percentage, protein percentage, electrical conductivity (both daily maximum and daily averaged), and SCS were the traits evaluated (in the following equation these traits are generalized as y). The matrix model used for each different y was

y=xβ+Zα+e

where y = the vector of the observations of the dependent variable; β = the vector of the fixed effects, associated with y through the incidence matrix of X; α = the vector of the random effects and additive genetic value of the animal, associating a with y through the incidence matrix Z; and e = the vector of the residual effects.

Both parity (multiparous subset only) and DIM were converted to categorical variables by grouping the observations. Specifically, parity had two levels (i.e., second and third parity in the first group and more than three parities in the second group), while DIM were grouped into five classes of 60 days (from 5 to 64 days, from 65 to 124 days, from 125 to 184 days, from 185 to 244 days, from 245 to 305). The DIM classification was performed, on the one hand, to reflect the phases of lactation (i.e., early lactation: 5–64 DIM; mid lactation: 65–124 DIM; late lactation: DIM > 125). We retained only data from the 5th to the 305th DIM to completely exclude the initial colostrum phase and to limit the lactation to the conventional 305-day lactation used for selection purposes.

On datasets 1 and 2, the Kolmogorov–Smirnov test was performed to verify the normal distribution for all the traits and choose the analysis to be used to compare the means of the traits measured by AMS and OMR. The Kruskal–Wallis test was then performed to verify if a difference existed between the populations measured by AMS and OMR.

## 3. Results

### 3.1. Comparison of Datasets 1 and 2

Phenotypic traits collected by AMS and OMR are summarized in Table 1. Only MY and protein percentage measured by AMS were normally distributed and SCS did not show a normal distribution even after logarithmic transformation of SCC. Furthermore, in the subsequent analysis, SCS was preferred to SCC because its distribution was more similar to the normal distribution than SCC. Considering the whole datasets (dataset 1 versus dataset 2 and AMS versus OMR data in dataset 3), no statistical differences were observed between traits measured by AMS and OMR (Table 1), despite the different sampling and measurement method.

**Figure 1:**
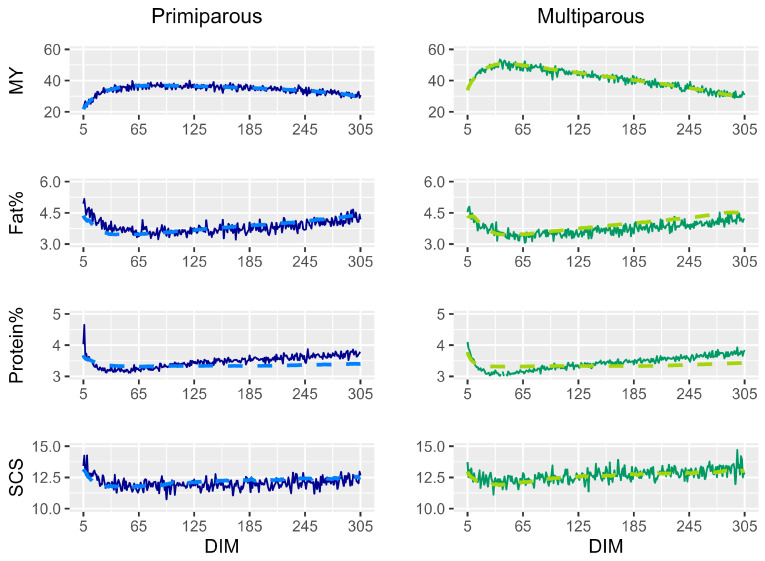
Graphical representation of the trend of production traits and SCS measured by automatic milking system (AMS—dashed lighter line) and official milk recordings (OMRs—continuous darken line) during lactation in primiparous (blue) and multiparous (green) cows.

The means of all traits measured, considering the DIM, are presented in Figure 1 to show the production curves of the analyzed animals, considering primiparous and multiparous cows separately. As expected, the lactation curve and the SCS were higher in multiparous than primiparous cows, whereas for fat and protein the curves are similar among parities.

The correlation between AMS-measured traits was also calculated and the results are shown in Supplemental Table S1. MY has a negative correlation with fat (−0.42, *p*-value < 0.001) and protein percentage (−0.17, *p*-value < 0.001), and a positive correlation with aEC (0.20, *p*-value < 0.001) and mEC, although almost null (0.03, *p*-value < 0.001). Furthermore, mEC was not correlated with the production traits, whereas aEC showed a positive correlation with MY (0.20, *p*-value < 0.001) and a low negative correlation with fat (−0.08, *p*-value < 0.001) and protein percentage (−0.13, *p*-value < 0.001). Lastly, the SCS showed a negative correlation with MY (−0.17, *p*-value < 0.001) and a positive correlation with the other traits, namely fat (0.23, *p*-value < 0.001) and protein percentage (0.15, *p*-value < 0.001), mEC (0.16, *p*-value < 0.001), and aEC (0.05, *p*-value < 0.001).

### 3.2. Comparison of AMS and OMR Data in Dataset 3

Correlations between the same traits measured by AMS and OMR were calculated for the whole herd and then stratified by parity (Table 2).

**Figure 2:**
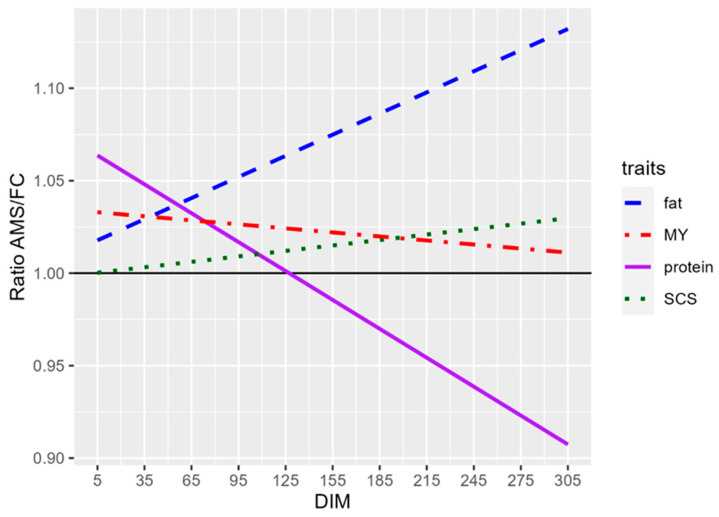
Linear regression of the ratio of data measured by automatic milking system and official milk recording (AMS/OMR, dataset 3) for the production traits and SCS.

Furthermore, the ratio between AMS and OMR values was calculated for each measurement of all the traits across the DIM, in order to evaluate whether AMS measurement errors were random or systematic and whether these errors changed throughout lactation. To better understand the pattern of these ratios, linear regressions were calculated using DIM as the independent variable and the AMS/OMR ratio as the dependent variable and the resulting trends are shown in Figure 2.

The linear regressions for MY and SCS were close to Y = 1 (the black horizontal line), suggesting that AMS measurements were alternately higher and lower than OMR, with no consistent trend. In contrast, the regressions for fat and protein percentages showed distinct slopes. The fat percentage regression had a positive slope with an intercept of 1020, suggesting that the AMSs tended to overestimate fat content, especially in late lactation. The protein percentage regression had a negative slope with an intercept of 1067, crossing the Y = 1 line at approximately 130 DIM, indicating a shift from overestimation to underestimation as lactation progressed.

### 3.3. Heritabilities

The heritabilities of all the traits were calculated using dataset 1 and 3. As for dataset 1, we analyzed 885,969 observations from 2181 cows (392.37 ± 293.97 observation per animal) distributed among five farms (451.80 ± 242.70 animals per farm). The cows’ mean age at test day was 3.65 ± 1.37 years. The mean parity of the cows was 2.07 ± 1.18, with values ranging from 1 to 9. Regarding DIM, the observations were quite evenly distributed between the five classes, with an average number of observations per class of 177,194 ± 18,404 (range: 148,997–196,338).

Heritability was estimated for the entire lactation period using test-day data from the AMSs and the results are summarized in Table 3. Heritability was estimated for primiparous and multiparous cows separately, since heritability often has higher values for multiparous cows. In the present study this was true for all of the traits, except for MY and protein percentage, where heritability was higher in primiparous cows (0.38 versus 0.23 for MY and 0.48 versus 0.26 for protein percentage, respectively).

Heritability was also estimated during the lactation for period of 60 days, as described above, to uncover the effects that different factors have on the measured traits during lactation. Reducing the period of DIM over which heritability is estimated consequently reduces the environmental effects and increases the genetic effect. Thus, these heritability estimates were higher than the ones for the entire lactation for almost all of the five classes of 60 days (Table 3). Furthermore, heritability estimates increased during lactation for all the traits in primiparous cows, whereas for multiparous cows we obtained the highest values from 125 to 184 DIM for almost all the traits.

Moreover, heritability was estimated for all the traits using data from dataset 3. The results are summarized in Table 4. Milk-production traits showed higher heritability in AMS than OMR data, whereas SCS heritability was higher for OMR data.

## 4. Discussion

Automatic milking systems are considered the key to technological advancement in dairy farming [19]. Milking robots collect data on numerous traits, including animal health-related status variables, like EC and SCC, and milk-productive traits like fat and protein percentage and MY. The analysis of the correlation between milk traits estimated using AMS data showed that the correlation of fat and protein percentage with SCS (0.23 and 0.15, respectively) is comparable to that found in the literature [20,21]. As widely reported in the literature, MY showed a negative correlation with fat percentage, protein percentage, and SCS (respectively, −0.42, −0.17, −0.17), which is consistent with previously published results [22]. The relationships between electrical conductivity and the other measured traits vary substantially depending on whether aEC or mEC is considered. In particular, mEC showed an almost null correlation with the MY (0.03, *p*-value < 0.001), fat percentage (0.00, *p*-value = 0.11), and protein percentage (−0.06, *p*-value < 0.001), but a higher correlation with the SCC (0.22, *p*-value < 0.001) and SCS (0.16, *p*-value < 0.001). This suggests that mEC is more influenced by salt concentration (which significantly varies during mastitis [23]) than it is influenced by fat or protein percentage. This justifies a stronger association of mEC with high SCC, which is usually related to mastitis. Nevertheless, the correlation remains relatively weak, possibly due to the fact that somatic cell count (SCC) tends to increase more markedly than electrical conductivity (EC) during mastitis events. On the contrary, aEC showed a lower correlation with SCC (0.10, *p*-value < 0.001), SCS (0.05, *p*-value < 0.001), and fat percentage (−0.08, *p*-value < 0.001), but a greater correlation with MY (0.20, *p*-value < 0.001) and protein percentage (−0.13, *p*-value < 0.001). In our opinion, based on the results of this study, mEC appears to be a more useful trait than aEC for mastitis management.

AMSs have the potential to be used as phenotypic records for breeding selection, provided that the measurements are accurate and comparable to official methods. In Italy, OMR is carried out monthly, measuring MY, fat percentage, protein percentage, and SCC. Our results demonstrated that the means of the traits measured by AMS data were not statistically different from the data coming from the same population measured by OMR, as shown in Table 1. Nonetheless, when comparing the data of the individual cows registered by AMS and OMR in the same day (Table 2), the correlation between AMS and OMR was moderate for all the traits, with the only exception being MY (0.931, *p*-value < 0.001).

Considering the correlations of fat percentage (0.517, *p*-value < 0.001) and protein percentage (0.477, *p*-value < 0.001), the lower values are probably due to differences in how the robot measures the traits. AMSs do not use the official method’s technology. Furthermore, since for OMRs the milk analysis is performed on a singular sample taken during one of the daily milkings, whereas AMS data are the average of all the daily measures (usually more than two measures per day), AMS data are less affected by fluctuations due to the circadian rhythm of the cows and this could further amplify the differences.

The correlation of SCC between AMS and OMR data collection methods was higher (0.615, *p*-value < 0.001), suggesting that AMSs correctly identify the SCC trend. This finding is particularly relevant given that the trait is theoretically more prone to variability introduced by sampling conditions. In fact, both AMSs and OMRs analyzed one daily sample. As a matter of fact, in AMSs the frequency of the SCC measurements is not regular, but is decided by the farmer, and in the farms selected for this study SCC was usually measured once every three milking events. Moreover, when the SCC measure is higher than 200,000, the AMSs will start measuring the SCC at each milking and the reported AMS data is the average of all the milkings of the day. Thus, the sample taken during the OMRs in the majority of cases is different from the one provided by AMS. Furthermore, SCC has a circadian trend and different milkings during the same day have physiologically different SCCs and patterns [24,25]. Fat percentage and protein percentage also possess differences during the day, but, since AMS output consists of an average of fat percentage and protein percentage, the differences due to circadian variations are reduced.

Despite the moderate values of the correlations between AMS and OMR data, the means of SCS during lactation (Figure 1) were almost overlapping and the differences were due to peaks in the OMR data that were, for each DIM, about thirty times less than that of AMS, whose means were more linear. Obviously MY during lactation had a much more similar trend, as expected by the high correlation between AMS and OMR data. On the contrary, the means of fat percentage and protein percentage during lactation measured by AMS and OMR did not overlap as much.

To assess whether the AMSs overestimated or underestimated each trait, we calculated the AMS-to-OMR ratio for test day data and evaluated its linear regression on DIM. These linear regressions confirmed the differences between AMS and OMR data as observed in Figure 2. The AMSs overestimated the fat percentage throughout the whole lactation period and the ratio increased according to the DIM. For the protein percentage, on the contrary, the ratio decreased during the lactation and for about the first 125 days the AMSs overestimated the protein percentage and then underestimated it. The ratio of the SCC and SCS is regular throughout the lactation period and the mean is equal to 2.13 for the SCC and 1.01 for the SCS. For both traits the median is almost equal to 1 (1.13 for the SCC and 1.01 for the SCS) and the first quartile is 0.52 for the SCC and 0.93 for the SCS and the third is 2.38 for the SCC and 1.11 for the SCS. Therefore, the measurements were equally distributed between over- and under-estimated values. Furthermore, in 25% of cases, the AMSs measured twice the SCC than the OMRs, while in 25% of cases it was the other way around. Considering that the SCC was below 200,000 in 86.5% of the cases and below 400,000 in 91.9%, the observed differences at low SCC levels were likely due to daily fluctuations or to the previously mentioned differences in AMS and OMR sampling. Only 0.07% of the data showed a large discrepancy between AMS and OMR (a ratio greater than 10 or less than 0.1), which may be explained by the non-simultaneous sampling or by occasional analytical errors. Since the discrepancies between AMS and OMR are mainly relevant when the SCC values fall between 200,000 and 400,000 and only about 5% of our data falls in such range and may have been significantly affected by the differences between the two measurement systems, for the remaining 95% of the AMS data the SCC values are a useful indicator of the health status of the cows. Thus, given the large number of measurements performed by AMS, the SCC values derived from the AMS data can be considered reliable. On the contrary, in its current state, AMS fat percentage and protein percentage should be considered reliable with caution and are not yet accurate enough to be used for selection. As reported in the literature [15], since their initial introduction, AMSs have encountered several substantial challenges, including the development of more efficient and less disruptive milking arms and improvements in milking hygiene. Adequate teat sanitation is not always ensured in AMSs, and research continues to refine the cleaning procedures. Significant progress in sensing technologies is still ongoing and machine learning approaches for predictive modeling are increasingly being integrated into these systems. Consequently, further improvements in measurement accuracy and prediction performance are expected in the near future. At present, AMSs allow the integration of externally obtained analytical data for sensor calibration and improved measurement accuracy. In the farms involved in this study, this option was not properly utilized. Bulk milk data was only entered occasionally by a few farmers and data on individual animals was never entered. Therefore, the accuracy could improve if values from official analyses related to individual animals were consistently entered and the incorporation of OMR data should be encouraged.

The heritability was estimated for all traits using AMS data for primiparous and multiparous cows, to verify if heritability changes after the first lactation, as suggested by scientific literature [5]. In addition, heritability was also estimated by dividing the lactation into periods of 60 days to see how values changed during lactation.

To the best of our knowledge, only one study can be found in the scientific literature [26] in which the authors estimated the heritability of fat and protein percentages using AMS data, while a second study used fat and protein yields instead of percentages [17]. Sitkowska et al. (2024) obtained estimates of 0.36 and 0.28 for fat percentage and protein percentage, respectively [26]. These results were consistent with the results of the present study, where the heritability results were similar to what already reported, since we divided the population into primiparous and multiparous cows both for fat percentage (0.34 and 0.32, respectively) and for protein percentage (0.36 and 0.24, respectively). On the contrary, Nixon et al. (2009) found lower values both for fat yield (0.20) and protein yield (0.21) [17]. Higher heritability values for fat and protein percentage than for yield were also described in studies that used milking parlor data and, in particular, values of 0.53–0.55 were described for fat percentage and of 0.35–0.37 for fat yield, or of 0.56–0.59 for protein percentage and of 0.39–0.42 for protein yield [27]. It should be noted that, as reported in the scientific literature [27,28,29], the range of variation in heritability estimates from non-AMS data shows substantial variability (0.24–0.66 for fat percentage and 0.40–0.69 for protein percentage). Our results are also consistent with the ones obtained by Buitenhuis and Poulsen (2023) on milking parlor data (0.33–0.38 for fat percentage and 0.40–0.43 for protein percentage) [29].

Heritability of MY was 0.29 for primiparous and 0.18 for multiparous cows, similar to the result obtained by Nixon (2009) [17] of 0.26, an intermediate value that is probably due to the fact that they used data from the entire herd, whereas this study divided the herd into primiparous and multiparous cow groups for its estimation and consequentially can better describe the difference among parities. Instead, Sitkowska et al. (2024) found lower heritability for MY, namely 0.12 [26]. In comparison, Pedrosa et al. (2023) estimated daily MY heritability during the lactation period using AMS data and their results ranged between 0.07 and 0.28, with heritability increasing during lactation [8]. We also found a similar crescent trend in our study for primiparous cows (from 0.16 to 0.38), even though our estimation was made using data divided into periods of 60 days; instead, for multiparous cows, the trend was different, with a peak between 65 and 184 DIM (0.20) and a lower value for the last period (0.06). It has to be noted that the lower values from Pedrosa et al. (2023) were obtained in the first days of lactation, where many environmental factors are involved [8].

Considering the entire lactation period, heritability of conductivity traits was 0.164 and 0.093 for aEC and 0.077 and 0.088 for mEC for primiparous and multiparous cows, respectively. The value of aEC ranged from 0.11 to 0.32 for primiparous cows, while for multiparous cows the model is unable to produce a reliable estimate, whereas values of mEC ranged from 0.05 to 0.10 for primiparous cows and from 0.06 to 0.13 for multiparous cows (Table 3). Pedrosa et al. (2023) estimated EC heritability values ranging from 0.38 to 0.49 during the lactation period, values that are higher than the ones found in the present study [8]. This could be due to the differences in the specific EC measurement systems used in the two studies. To our knowledge, in only one study were similar values found, namely 0.37–0.46 for the single quarter and 0.53 for the average of the quarters [30], but data were taken for a period of only 30 days. As in our study, Wethal et al. (2020) found a higher heritability for aEC (0.35) than for mEC (0.12 to 0.27 during lactation), even if our heritability estimates were lower [31]. Furthermore, for Wethal et al. (2020) heritability decreased during lactation while in the present study the trend changes: aEC increased for primiparous cows and decreased for multiparous cows; mEC increased during the lactation for primiparous cows, while it remained stable for multiparous cows during the whole lactation [31]. Samaraweera et al. (2022) found low EC heritability (0.02–0.04) that was similar to our mEC values [7]. Maximum EC heritability was lower both in the present study and in the literature, indicating that it is more influenced by environment than aEC. Furthermore, even if all these heritability estimates came from AMS data, the population analyzed and how the EC was recorded makes it difficult to compare the results. Our results included EC for traits with low heritability, and therefore its use in selection could give slow and unstable results.

Lastly, the SCS heritability was 0.06 for primiparous (the value remains stable at 0.04 during the lactation period with two lower values at 65–124 and 245–305 DIM) and 0.06 for multiparous cows (this value increased during the lactation from 0.05 to 0.09 at 125–184 DIM and decreased in the last 60 days to 0.07). These heritability values are similar to those already reported in the literature: 0.01 to 0.08 [32]; 0.13 [33]; 0.06 [20]; and 0.02 to 0.08 [8]. Particularly, Pedrosa et al. (2023) showed a similar trend in SCS during the lactation period [8]. Heritability of the SCS measured both from AMS data and OMR is low and therefore such a trait is overall considered a low-heritability trait, as it is influenced by numerous environmental factors (both intrinsic and extrinsic to the animal) such as the specific pathogen involved, the environmental conditions and the potential stressors affecting the animal, which are difficult to control for better estimation. Although the low heritability of SCS limits the potential genetic gain obtainable through selection, SCS is already the most used trait for selection of mastitis-resistant cows. Thus, the high frequency of AMS recordings provide valuable information for monitoring udder health, supporting timely management interventions, and selecting resistant cows, since it allows monitoring of the progression of mastitis. Moreover, the possibility to continuously track EC, particularly mEC, offers an additional indicator of mastitis status that could be combined with the SCC to enhance the accuracy of health assessments and early-detection and selection strategies.

Heritability was also estimated for the OMR and AMS data from dataset 3. The heritability estimates from the AMS data taken in proximity with OMR were similar to ones obtained using the whole AMS database, but were usually lower (Table 4). Since database 1 had a higher number of observations per animal than database 3, the heritability value obtained from the first database should theoretically be more accurate than the heritability estimated from data from database 3.

Daily monitoring of the various traits allows a better phenotypic characterization, which translates into a more accurate estimation of heritability. The values of heritability calculated using AMS data from database 1 were as follows: 0.34 and 0.32 for fat percentage, 0.36 and 0.24 for protein percentage, 0.29 and 0.18 for MY, and 0.06 and 0.06 for SCS, for primiparous and multiparous cows, respectively. Heritability values calculated using OMR data from dataset 3 were as follows: 0.13 and 0.12 for fat percentage, 0.26 and 0.23 for protein percentage, 0.30 and 0.16 for MY and 0.15 and 0.14 for SCS, for primiparous and multiparous cows, respectively. When focusing on the traits most reliably recorded by AMSs (i.e., the MY and the SCS), it is noteworthy that heritability estimates were higher for the MY and lower for the SCS when measured by AMS compared to OMR. These results suggest that the huge amount of data from AMSs could reduce the environmental impact on the MY variance. On the other hand, the lower values for SCS suggest that the environmental factors affecting the SCS are better captured with a larger amount of data, and the small amount of data from OMRs probably does not optimally describe the entire SCS trend during the entire lactation period. Given these results, using data from AMSs would allow better selection of animals, despite the lower heritability.

## 5. Conclusions

Regarding production traits, the moderate correlation between AMS and OMR data for fat and protein percentage, that was probably due to low AMS precision, demonstrates a low reliability of AMS measures. This low reliability advises against their use for selection, despite the high heritability value. As it is possible to input data from OMR or other analysis conducted on milk samples to the robots’ software for calibration purposes, it would be possible to improve data reliability and its applicability for selection.

As for the SCC, since the mild correlation is probably due to the different sampling methods, AMS data is able to identify animals with a low SCC and monitor the evolution of the trait daily and can, therefore, be considered useful for phenotypic evaluation. Unfortunately, SCS showed a low heritability, suggesting the need for careful use in selection. Similarly, EC also showed low heritability. However, since the mEC shows a greater correlation than the aEC with the SCC (0.22 versus 0.10) and SCS (0.16 versus 0.05), it seems a more interesting trait. An index derived by the union of the two traits could be a more precise and useful tool for the selection of resistant animals from AMS data.

## Figures and Tables

**Table 1:** Comparison between the same traits measured by automatic milking system (AMS) (dataset 1) and by official milk recording (OMR) (dataset 2) and between the intersection of the two datasets (dataset 3). Mean, standard deviation, and sample size are reported. As electrical conductivity (EC) was not measured in the OMRs, only AMS data are shown. The Kruskal–Wallis test did not show significant statistical differences and therefore the *p* value was not reported.

Traits	AMS (Dataset 1)	OMR (Dataset 2)	AMS (Dataset 3)	OMR (Dataset 3)
Milk Yield (Kg)	(n = 885,969) 39.08 ± 10.37	(n = 33,021) 37.17 ± 10.53	(n = 19,488) 39.26 ± 10.21	(n = 19,488) 38.85 ± 10.44
Fat (%)	(n = 877,825) 3.88 ± 0.69	(n = 33,021) 3.85 ± 0.90	(n = 19,488) 3.88 ± 0.67	(n = 19,488) 3.77 ± 0.92
Protein (%)	(n = 877,825) 3.51 ± 0.22	(n = 33,021) 3.48 ± 0.4	(n = 19,488) 3.36 ± 0.22	(n = 19,488) 3.42 ± 0.37
mEC ^1^	(n = 885,948) 70.37 ± 11.31	-	-	-
aEC ^2^	(n = 885,948) 67.62 ± 6.84	-	-	-
SCC (K ^3^/mL)	(n = 779,213) 166.7 ± 370.86	(n = 32,398) 227.4 ± 675.04	(n = 17,350) 162.7 ± 359.15	(n = 17,350) 234.2 ± 760.63
SCS	(n = 779,213) 12.39 ± 1.85	(n = 32,398) 12.51 ± 1.89	(n = 17,350) 12.4 ± 1.82	(n = 17,350) 12.45 ± 1.92

^1^ mEC = maximum electrical conductivity; ^2^ aEC = average electrical conductivity; ^3^ K = 1000 cells.

**Table 2:** Correlation between traits measured by automatic milking system (AMS) and functional control (OMRs) (dataset 3) for the whole dataset, and then specifically for primiparous and multiparous cows.

AMS vs. OMR	Total Herd	Primiparous	Multiparous
MY	0.931 ***	0.914 ***	0.922 ***
Fat percentage	0.517 ***	0.530 ***	0.535 ***
Protein percentage	0.477 ***	0.467 ***	0.491 ***
SCC	0.615 ***	0.582 ***	0.618 ***
SCS	0.494 ***	0.582 ***	0.618 ***

*** *p*-value < 0.001.

**Table 3:** Heritability estimates of productive- and health-related status traits obtained from automatic milking system (AMS) data considering the whole lactation period (5–305 DIM) and five consecutive periods of 60 days each.

		5–305 DIM		5–64 DIM (I)		65–124 DIM (II)		125–184 DIM (III)		185–244 DIM (IV)		245–305 DIM (V)	
		h^2^	SE	h^2^	SE	h^2^	SE	h^2^	SE	h^2^	SE	h^2^	SE
Primiparous	Milk yield	0.293	0.042	0.163	0.040	0.291	0.056	0.379	0.061	0.371	0.064	0.364	0.063
	Fat percentage	0.341	0.035	0.307	0.039	0.453	0.051	0.513	0.053	0.490	0.057	0.387	0.061
	Protein percentage	0.355	0.039	0.261	0.039	0.455	0.055	0.501	0.058	0.488	0.060	0.400	0.061
	aEC	0.164	0.034	0.111	0.031	0.235	0.046	0.215	0.048	0.317	0.054	0.249	0.055
	mEC	0.077	0.016	0.053	0.015	0.086	0.024	-	-	0.097	0.024	0.100	0.026
	SCS	0.061	0.016	0.041	0.019	0.019	0.017	0.042	0.022	0.040	0.027	0.029	0.026
Multiparous	Milk yield	0.184	0.035	0.105	0.032	0.191	0.041	0.200	0.044	0.117	0.042	0.063	0.038
	Fat percentage	0.321	0.035	0.211	0.033	0.388	0.045	0.496	0.048	0.468	0.050	0.317	0.055
	Protein percentage	0.241	0.032	0.171	0.031	0.320	0.044	0.384	0.047	0.405	0.052	0.407	0.057
	aEC	0.093	0.034	0.139	0.040	-	-	-	-	-	-	-	-
	mEC	0.088	0.017	0.103	0.017	0.086	0.021	0.095	0.023	0.060	0.026	0.129	0.033
	SCS	0.064	0.018	0.045	0.018	0.056	0.023	0.085	0.028	0.057	0.027	0.068	0.029

**Table 4:** Heritability estimates of productive- and health-related traits obtained from automatic milking system (AMS) and official milk recording (OMR) data on the common dataset (dataset 3).

		AMS		OMR	
		h^2^	SE	h^2^	SE
Primiparous	Milk yield	0.283	0.048	0.275	0.044
	fat percentage	0.363	0.043	0.143	0.028
	protein percentage	0.395	0.045	0.310	0.038
	SCS	0.067	0.019	0.130	0.037
Multiparous	Milk yield	0.252	0.039	0.219	0.036
	fat percentage	0.424	0.043	0.193	0.028
	protein percentage	0.340	0.041	0.252	0.035
	SCS	0.070	0.020	0.155	0.032

## Data Availability

The datasets used and/or analyzed during the current study are available from the corresponding author on reasonable request.

## Supplementary Materials

The following supporting information can be downloaded at: https://www.mdpi.com/article/10.3390/ani16010001/s1, Table S1: Correlation between traits collected by automatic milking system (AMS).

## Abbreviations

The following abbreviations are used in this manuscript:

AMS	Automatic milking systems
EC	Electrical conductivity
mEC	Maximum electrical conductivity
aEC	Average electrical conductivity
OMR	Official milk recording
MY	Milk yield
MF	Milk flow
