# Supplementary material for: Assessing the Reliability of Automatic Milking Systems Data to Support Genetic Improvement in Dairy Cattle
Source: Animals (Basel). 2025 Dec 19;16(1):1. doi: 10.3390/ani16010001 (PMC12785074; doi:10.3390/ani16010001)
Supplement: Supplementary file 1 [file animals-16-00001-s001.zip › animals-4010561-supplementary.pdf]

Supplemental Table S1. Correlation between traits collected by automatic milking system (AMS).

	Fat percentage	Protein percentage	mEC	aEC	SCC (K/ml)	SCS
MY	-0.42 ***	-0.17 ***	0.03 ***	0.20 ***	-0.12 ***	-0.17 ***
Fat percentage	-	0.26 ***	0.00	-0.08 ***	0.11 ***	0.23 ***
Protein percentage		-	-0.06 ***	-0.13 ***	0.02 ***	0.15 ***
mEC			-	0.62 ***	0.22 ***	0.16 ***
aEC				-	0.10 ***	0.05 ***
SCC (K/ml)					-	0.68 ***

** p-value <0.01
*** p-value <0.001
